# The Georgia Association

**Published:** 1891-04

**Authors:** 


### THE GEORGIA ASSOCIATION.

We desire to call the attention of our readers to the letter of Secretary Moore which appears elsewhere in this issue. We would urge all members of the Association who can do so to attend the Augusta meeting and participate in its deliberations; and we would also urge all Georgia doctors not already members of the Association to go down and unite with it at once. It is not necessary for us to remark on the great good which is accomplished at these annual meetings.

We of the South are too often reproached with the fact that we do not possess that energy and activity which is manifested by our northern neighbors. This is true of southern doctors, as well as southern merchants and southern farmers. The medical societies of the North are numerous and well attended. Members are eager to record their experiences and discuss the experiences and reported cases of others. The advantages of this spirit are obvious; it blesses him that gives and him that takes. This same spirit should pervade the profession throughout the South. To some extent, at least, the cases] occurring in the practice of our northern brethren, which are observed carefully, reported and discussed, can be duplicated in the experience of our own practitioners, yet, as a rule, the only record that is made of them is their entry in the physician's visiting list.

The statement was made last fall in this city, at the Surgical and Gynecological Association, by Dr. Joseph Price, of Philadelphia, who is easily among the best of American surgeons, that some of our best surgery is being done by country doctors. It is safe to say that three-fourths of this work is never recorded.

Manifestly, the only way of arriving at a just conclusion regarding any given disease or condition is by comparing recorded experiences.

The Journal will be much gratified if the above remarks will stimulate some of its readers to give others the benefit of their observations. Certainly its columns are always open for the purposes indicated.

